# Isotherms and Kinetic Studies of Copper Removal from Textile Wastewater and Aqueous Solution Using Powdered Banana Peel Waste as an Adsorbent in Batch Adsorption Systems

**DOI:** 10.1155/2023/2012069

**Published:** 2023-05-26

**Authors:** Momina Seleman, Takele Sime, Abate Ayele, Assefa Sergawie, Thabo Nkambule, Jemal Fito

**Affiliations:** ^1^Department of Chemical Engineering, College of Biological and Chemical Engineering, Addis Ababa Science and Technology University, Addis Ababa 16417, Ethiopia; ^2^Department of Environmental Engineering, College of Biological and Chemical Engineering, Addis Ababa Science and Technology University, Addis Ababa 16417, Ethiopia; ^3^Department of Biotechnology, College of Biological and Chemical Engineering, Addis Ababa Science and Technology University, Addis Ababa 16417, Ethiopia; ^4^Department of Industrial Chemistry, College of Applied Sciences, Addis Ababa Science and Technology University, Addis Ababa 16417, Ethiopia; ^5^Institute for Nanotechnology and Water Sustainability (iNanoWS), College of Science, Engineering and Technology, University of South Africa, Florida Science Campus1710, Johannesburg, South Africa

## Abstract

Heavy metals that are present in surface water and wastewater are becoming a severe environmental problem. Because of its toxicity, heavy metal removal has become the main priority for environmental concerns. Banana peels are low-cost agricultural waste that could be used for heavy metal adsorption in wastewater. The main objective of this study is to evaluate the effective powdered banana peel for the removal of copper (II) from aqueous solutions and real wastewater. The banana peels were collected from domestic waste and ground to get a particle size of 150 *µ*m. Powdered banana peel waste adsorbent (PBPWA) contained moisture content, ash content, volatile matter, and bulk density of 3.8%, 3.5%, 37.5%, and 0.02 g/cm^3^, respectively. The Fourier-transform infrared spectroscopy (FTIR) results showed that the alkyne, aldehyde, and amide functional groups were dominant in the powdered banana peel surface, and the scanning electron microscope showed the morphology of the adsorbent. Physicochemical characteristics of the raw wastewater revealed that the concentration of Cu (II), Pb (II), COD, BOD5, and Cd (II) were 2.75 mg/L, 2.02 mg/L, 612.16 mg/L, 185.35 mg/L, and 0.01 mg/L, respectively. At pH 5, adsorbent dose of 2g/100 mL, initial copper (II) concentration of 80 mg/L, and contact time of 90 min, the maximum removal efficiency of synthetic wastewater was 96.8% and textile wastewater was 69.0%. The adsorption isotherm fitted well with the Langmuir isotherm model at *R*^2^ = 0.99. The kinetics of copper (II) adsorption followed the second-order kinetic model better. Finally, these studies showed that banana peel bio-adsorbent is a potential adsorbent for heavy metal removal from synthetic and textile wastewater.

## 1. Introduction

Eco-toxicity as a result of polluted water sources' effects on living organisms has been a major concern for the last few decades [1–3]. As a result of rapid industry and extensive urbanization, waste effluents are released directly into the river water, degrading the ecosystem [1, 4]. Heavy metals are poisonous and non-biodegradable and tend to accumulate in living organisms even at low doses [5]. These metals can cause severe adverse effects on humans, animals, and plants and harm aquatic life [6]. Acute or chronic exposure of humans to these metals causes a range of health problems including cancer, blood pressure, paralysis, blood sugar, tumors, liver and lung failure, joint disease, brain haemorrhage, and sudden death [7]. The most common heavy metals found in industrial effluents include nickel, zinc, silver, lead, iron, chromium, copper, arsenic, cadmium, and uranium [8]. Copper, on the other hand, is commonly found in high amounts of effluent from metal finishing, electroplating, textiles, plastics, and etching [9] Furthermore, copper is a highly hazardous metal even at low concentrations; therefore, copper-contaminated wastewater must be cleansed before being discharged into the environment [9]. According to the United States Environmental Protection Agency (USEPA), copper ion concentrations of industrial effluents should not exceed 1.3 mg/L [10], while copper ion concentration of drinking water should not exceed 0.9 mg/L, according to the World Health Organization [11].

The development of novel separation techniques has resulted in the development of effective metal removal methods. Metals can be removed from aqueous solutions using traditional methods. Water treatment methods include precipitation, ion exchange, flocculation, adsorption, electrochemical processes, electrodialysis, nanofiltration, and reverse osmosis [12–15]. When heavy metals are present in low concentrations, however, these methods are either ineffective or costly, and they may also contribute to the generation of difficult-to-handle secondary wastes [16]. As a result, it is critical to reduce hazardous heavy metals to a level that is both cost-effective and environmentally responsible [13]. Adsorption is a new technology to remove heavy metals from different industrial wastewaters due to its low cost, local availability, and technological feasibility. Adsorption has recently emerged as a viable heavy metal removal alternative [17]. An adsorbent is a substance or material that has the ability or tendency to absorb other chemicals or materials [18]. Biosorbents are materials generated from biological sources that can absorb particular contaminants [19, 20]. Environmental scientists are very interested in this biological-origin product of environmentally friendly cleansing [21].

Heavy metal adsorption could gain from bio-adsorbents made from agricultural waste [12, 22, 23]. Agricultural waste contains cellulose, hemicellulose, lignin, pectin, and other functional groups. Current studies have reported many materials to be effective in removing Cu (II) in the solution in question [5, 24], including saw dust, pine fruit, rice husk, *Cercis siliquastrum* L. leaves, activated carbon, natural zeolite, and wheat straw [25]. However, banana is one of the most important fruits in Ethiopia. Banana fruit contains 30 to 40% of banana peel. Banana peel (BP) is the ordinary waste of banana fruit [26]. The banana waste contains high cellulose and minerals. A huge quantity of banana peel leads to major disposal problems as well as a massive waste of resources. BP surfaces have a different range of functional groups such as carboxyl, hydroxyl, and amide groups that have been proven to be crucial features in the biosorption processes [27]. BP is an attractive adsorbent due to its porous structure with various surface groups, availability, organic compound constituents, affordability, and environmental friendliness [28]. Currently, several researchers have used raw and chemically treated BP for the removal of heavy metals from industrial wastewater and aqueous solution. The objective of the present study was to investigate the utility of banana peel waste powder to serve as a biosorbent for the removal of copper from aqueous solution and textile wastewater by isotherm studies and kinetic studies. Additionally, several reaction variables that affect the removal percentage were investigated.

## 2. Materials and Methods

### 2.1. Materials

The copper stock solution (1000 mg/L) was prepared by dissolving 3.929 g of copper sulfate (CuSO_4_.5H_2_O) (Sigma-Aldrich, USA, 99.9%) in 1000 mL of distilled water and then using it for all experiments with the appropriate dilution. Banana peels were collected from domestic waste and used throughout the experiments.

### 2.2. Preparation of Adsorbent

The banana peels were collected and washed with tap water and then distilled water. The material was washed and cut into little pieces before being dried in the sun for three days. The dried banana peels were washed again with distilled water and dried for 24 hours in a hot air oven at 105°C. Finally, the dried material was finely crushed and sieved through a 150 mm fine-mesh sieve before being stored in plastic bottles for further study as shown in supplementary file.

### 2.3. Characterization of Banana Peel Waste Adsorbent (BPWA)

The moisture, volatile matter, ash, and fixed carbon content of the powdered banana peel were determined using ASTM (D 2866–2869), and the bulk density of the powdered banana peel was determined using the water displacement method [29]. Fourier-transform infrared spectroscopy (FTIR) was used to describe the functional groups of powdered banana peel waste adsorbent (PBPWA) (INSPECT F 50). The data were analyzed using standard software after the spectra were obtained between 4000 and 400 cm^−1^ (Origin 6.0). The surface morphology of the BPW adsorbent was observed using scanning electron microscopy (RAffinity-1S) before and after Cu treatment.

### 2.4. Collection and Characterization of Textile Industry Wastewater

Polyethylene bottles were used to collect samples. Before sampling, the bottles were cleaned in a hot water bath for 48 hours with a 10% (v/v) nitric acid solution. After that, they were washed and rinsed with distilled water. The wastewater was collected from the textile industry in Addis Ababa, Ethiopia. Using the composite sampling method, the samples were collected directly from the factory outlet of the dying section. The wastewater samples were collected three times on three different days: November 14th at 9:30 a.m., November 17th at 3:00 p.m., and November 19th at 9:30 a.m. The wastewater was collected from the industry outlet on three different dates to determine the concentration of heavy metals at different time intervals because the wastewater collected from the industry at once could not represent the entire wastewater because water consumption varies widely in the industry depending on the mill, processes, equipment used, and type of materials produced. The samples were transported to the Chemical Engineering Laboratory at Addis Ababa Science and Technology University. The samples were kept in a refrigerator at 4°C. The APHA (2010) method was used to characterize the heavy metals Cu (II) and Pb (II) and analyze physicochemical characteristics (BOD_5_, COD, turbidity, pH, and temperature) of the effluent before and after adsorption.

### 2.5. Batch Mode Adsorption Study

Batch equilibrium adsorption studies were used to evaluate the produced PBPWA for copper removal from synthetic solution and textile wastewater. Regarding the adsorption capacity of Cu (II), the effects of various process parameters such as adsorbent dose (1–2.5 g/100 mL), solution pH (3–9), contact time (30–120 min), and initial Cu (II) concentration (40–120 g/L) were studied at constant temperature of 25°C and agitation speed of 200 rpm. Batch experiments were carried out in a shaking incubator with 250 mL glass vials containing 50 mL copper solution and 250 rpm agitation. The filtration method was used to separate the solid and liquid phases at the end of each experiment. A UV-Vis spectrophotometer (Shimadzu 1800) was used to determine the remaining concentration of Cu (II) in the solution after adsorption on BPW adsorbent at 630 nm. The percentage removal of Cu (II) was calculated using equation (1) as described by [30], and the equilibrium adsorption capacity was calculated using equation (2):

(1) $$\begin{array}{c} \%\;Removal=\frac{C_{o}-C_{t}}{C_{o}}\times 100\text{,} \end{array}$$

(2) $$\begin{array}{c} Qe=\frac{\left(Ci-Cf\right)*V}{M}\text{,} \end{array}$$

where *C*_*o*_ is the initial adsorbate concentration and *C*_*t*_ is the final adsorbate concentration after time *t*, *Q*_*e*_ (mg/g) is the equilibrium adsorption capacity, *C*_*i*_ (mg/L) is the initial concentration of copper in the solution, *C*_*f*_ (mg/L) is the equilibrium concentration of copper in the solution, *V* (L) is the volume of the copper solution, and *M* is the mass of the adsorbent.

### 2.6. Determination of Adsorption Isotherms

The Langmuir model assumes monolayer biosorption on identical and energetically equivalent active sites without any interaction between adsorbed molecules [31] and is written as

(3) $$\begin{array}{c} \frac{Ce}{qe}=\frac{1}{q_{0}k}+\frac{C_{e}}{q_{0}}\text{,} \end{array}$$

where *C*_*e*_ is the adsorbate equilibrium concentration (mg/L), *q*_*e*_ is the mass of solute adsorbed per unit mass of the adsorbent, *q*_*o*_ is a constant related to the adsorption capacity (mg/g), and *K* is the experimental constant.

The Freundlich isotherm describes the multilayer biosorption with the non-uniform distribution of sorption sites over the heterogeneous surface along with interactions between adsorbed molecules [31] and is expressed by

(4) $$\begin{array}{c} \log q_{e}=\log\;K+\frac{1}{n}\log C_{e}\text{,} \end{array}$$

where *C*_*e*_ is the adsorbate equilibrium concentration (mg/L), *q*_*e*_ is the mass of solute adsorbed per unit mass of the adsorbent, *q*_*o*_ is a constant related to the adsorption capacity (mg/g), *K* is the experimental constant, and *k* and *n* are Freundlich constants representing adsorbent adsorption capacity and intensity of adsorption, respectively.

### 2.7. Adsorption Kinetics

The kinetics of metal adsorption was analyzed using kinetic models which were pseudo-first-order (5) and the pseudo-second-order (6) as follows [32]:

(5) $$\begin{array}{c} \frac{dq\;t}{dt}=k1\left(qe-qt\right)\text{,} \end{array}$$

(6) $$\begin{array}{c} \frac{dq\;t}{dt}={k2\left(qe-qt\right)}^{2}\text{,} \end{array}$$

where qe and qt are the amounts of adsorbate adsorbed (mg/g) at equilibrium and at any time *t* and *K*1 (min^−1^) and *K*2 (min^−1^) are the pseudo-first-order and pseudo-second-order rate constants, respectively.

## 3. Results and Discussion

### 3.1. Physicochemical Analysis of Powdered Banana Peel

Table 1 shows the results of an investigation into the physicochemical characteristics of powdered banana peel adsorbent using the ASTM standard. According to the physicochemical analysis data, PBPWA has a low ash content (3.5%), pH of 6.6, a high content of volatile matter (37.5%), a low moisture content (3.8%), a high content of fixed carbon (55.2), and a low bulk density (0.02 g/cc). The observed moisture content and volatile matter were lower than those obtained in [33], and the fixed carbon of powdered banana peel (PBP) was higher than that of the adsorbent prepared from Banana Empty Fruit Bunch and *Delonix regia* fruit pod [34]. When compared to the results obtained from banana (*Musa paradisiaca*) stalks, the ash content, bulk density, and pH values were lower [35]. The average volatile matter content and low ash content usually increase the solid yield of carbon and produce high fixed carbon [36]. Moisture and bulk density are lower, indicating that the adsorbents have a higher removal potential [37].

### 3.2. Fourier-Transform Infrared Spectroscopy (FTIR)

**Figure 1:**
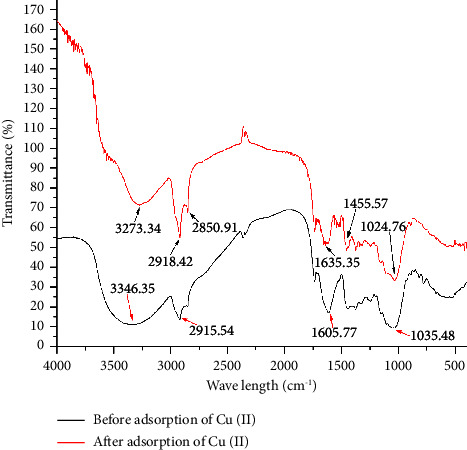
FTIR spectra of Cu (II)-treated (red) and Cu (II)-untreated (black) PBPWA.

Figure 1 illustrates the occurrence of noticeable differences in the FTIR peaks arising from the biomass of PBPWA before and after treatment with copper. The shifting of peak indicates that the banana peel biosorbent contributed to copper removal. The peaks region 3273.3 cm^−1^ indicate O-H groups [35], 2918.4 and 2850.9 are C-H stretch of Aldehydes [35], 1635.4 cm^−1^ is C=O stretching vibration in Esters [38], 1455.6 cm^−1^ is C-C=C stretching vibration in Esters (So Aromatic rings are shown by asymmetric stretch, while C-O stretch is indicated by 1035.5 cm^−1^ and 1024.8 cm^−1^. C-C stretching and C-N stretching both showed shifting [38]. The peaks markedly decreased probably due to the loss of polysaccharides during pyrolysis. The prepared powdered banana peels composed of lipids, proteins, crude fiber, and carbohydrates were recorded to identify the functional groups responsible for the Cu (II) metal ion coordination.

### 3.3. Scanning Electron Microscopy (SEM)

**Figure 2:**
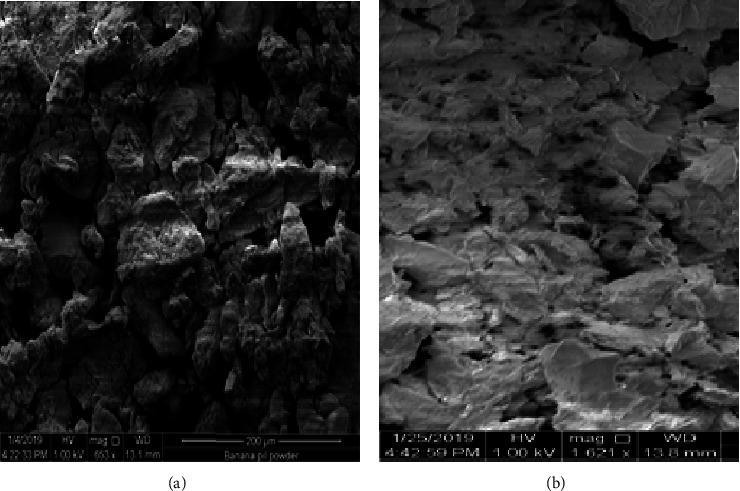
Untreated (a) and treated (b) PBPWA with Cu (II) solution.

Figure 2(a) indicates that before the metal was applied, the banana peel had a microporous structure with a heterogeneous rough surface with crater-like pores, and the particles were irregular in form with a micro-rough texture on their surface, which can aid in copper adhesion [2]. Cu-untreated BP powder showed a rough surface area with irregular crystal structure and a greater surface area per field, whereas the Cu-treated sample in Figure 2(b) had a smooth surface area and a reduced surface area due to Cu adsorption. The control group revealed a plain compact surface and wavy porous area, whereas the treated one showed smooth regions and the porous region was filled with copper metal ions. A possible reason for this behavior is the physicochemical interaction between the heavy metal ion and the functional groups on the surface of the banana peel [2].

### 3.4. Optimization of Parameters during Cu (II) Adsorption

#### 3.4.1. Effect of pH on Removal of Cu (II)

**Figure 3:**
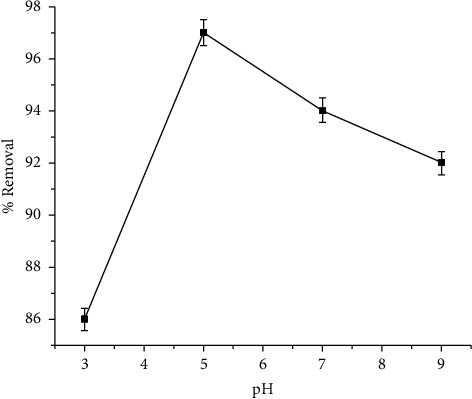
Effect of pH on the removal of Cu (II).

The surface characteristics of adsorbents, the ionic state of functional groups, and the species of metals are all affected by pH [2]. As the pH value increases, the percentage removal of Cu (II) increased from 86% to a maximum value of 97% at pH 5 and then decreased beyond 5, reaching a value of 92% at pH 9 as shown in Figure 3. Therefore, pH 5 was selected as the maximum pH for adsorption studies. At a low pH value, there is a high concentration of hydrogen ion, which competes with Cu (II) for adsorption on the binding sites of the adsorbent. As the pH rises from 3 to 5, the concentration of hydronium ions reduces, allowing Cu to adsorb more readily (II). As a result, the efficiency of removal increases. Cu (II) begins to precipitate as metal hydroxide (CuSO_4_.5H_2_O) attains pH above 5, reducing the availability of Cu (II) in the solution. As a result, the efficiency of removal decreases. The pH influence on Cu (II) removal efficiency followed a similar trend as copper adsorption onto other adsorbents such as laterite [39], rice husk [40], and tea residue ash [41].

#### 3.4.2. Effect of Time on the Removal of Cu (II)

**Figure 4:**
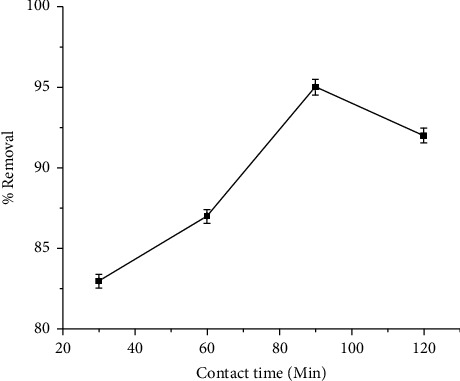
Effect of contact time on the removal of Cu (II).

Copper removal increased slowly as the contact time increased. However, after reaching 86% (Figure 4), copper removal increased rapidly with increasing contact time. However, after reaching 86%, the removal efficiency would decrease. Copper adsorption on PBPWA can occur in three distinct phases. Copper sorption gradually increased in the first phase. This could be caused by copper migration from the boundary layer to the internal pore. Rapid adsorption occurs in the second phase, and the total adsorption process takes 90 minutes. This could be due to the immediate sorption of copper on the PBPWA surface. Because the active site was unavailable in the third phase, copper sorption was reduced. Although Kandile and Nasr [42] found that there is no additional improvement in copper adsorption after 90 minutes, an equilibrium time of 90 minutes was chosen for all the following tests. According to Hossain et al. [2], the banana peel contains a large number of vacant active binding sites, and as a result, a large number of copper ions are rapidly bound to the banana peel. Because adsorption is the result of physical interaction (adhesion) between the adsorbate and the adsorbent, contact time is one of the influencing factors of the adsorption process [30].

#### 3.4.3. Effect of Adsorbent Dose on the Removal of Cu (II)

**Figure 5:**
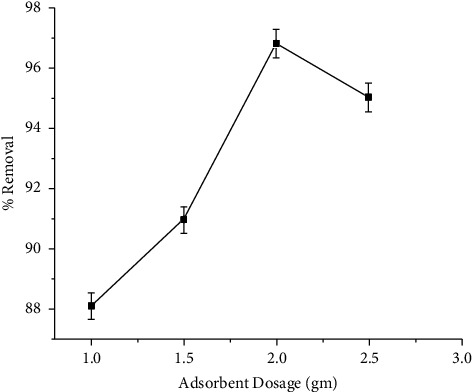
Effect of dose on the removal of Cu (II).

The copper removal increased from 88.1 to 96.8% when the adsorbent dose was raised from 1 to 2 g/100 mL, as shown in Figure 5. This progressive increase is attributed to an increase in surface area, which encouraged copper sorption. According to the surface site heterogeneity model, at high adsorbent doses, the surface is made up of sites with a spectrum of binding energies. The availability of higher energy sites reduces as a substantial fraction of lower energy sites are filled, resulting in a poor adsorption capacity [43]. The increase is linear since there are more vacant sites available for adsorption [44]. Lower adsorbent doses, on the other hand, expose all sorts of sites, and surface adsorption is saturated more quickly, implying a higher adsorption capacity. Increased adsorption site availability could lead to increases in the percent removal of adsorbate ions with increasing adsorbent dosage. The percent removal remained constant because the available adsorption sites were saturated at equilibrium [30].

#### 3.4.4. Effect of Initial Concentration on the Removal of Cu (II)

**Figure 6:**
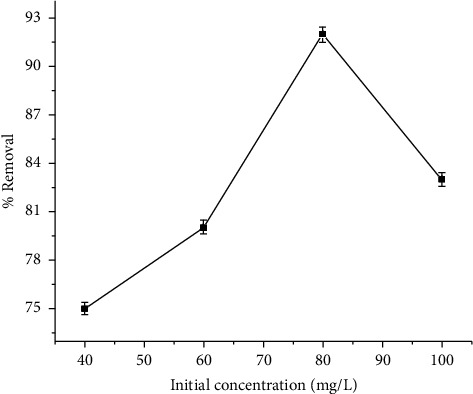
Effect of initial concentration on the removal of Cu (II).

Figure 6 shows the influence of initial copper concentration on the copper removal efficiency. Copper removal efficiency declined as the initial copper concentration increased, reaching equilibrium at 80 mg/L. The reason for this is that as the initial copper concentration rises, the capacity of the adsorbent materials decreases rapidly [45]. This is most likely because the total available adsorption sites for a fixed adsorbent dose were limited, and they became saturated at higher concentrations. According to Kumari's study [46], when the initial concentration of lead (II) increases from 10 mg/l to 100 mg/l, the percentage of lead (II) adsorption by banana peels decreases. This result confirms the findings of Taib et al. [47] who found that watermelon peels removed 83.0% of the Cu (II) ion at 70 mg/L and then started to remove 45.6% of it at 250 mg/L. The rapidly filled binding sites and saturating sorption sites on the surface of the adsorbent may be responsible for a decrease in removal efficiency at higher initial concentrations [7, 38, 48].

#### 3.4.5. Textile Industry's Treated and Untreated Wastewater Characteristics

Table 2 shows the characteristics of untreated and treated wastewater collected from the textile industry. Except for pH and temperature, the concentrations of most parameters in the untreated wastewater exceeded the standard. The concentration of Cu (II) ions, in particular, was higher than the standard; thus, it must be removed to avoid environmental risks. Due to the presence of dyes and additives used (such as caustic soda, sodium carbonate, and salts) during the textile manufacturing steps [49], Cu (II) ion occurs in textile effluent [50]. Cu is crucial for plants and microbes in a very small concentration, but in high concentrations, the metal is toxic [51]. In human beings, the metal is toxic, and it can cause Wilson's disease, hepatocellular degeneration, necrosis, brain damage, and death [52]. It affects plants' physiological activities such as photosynthesis, gaseous exchange, and nutrient absorption; thus, it causes reduction in plant growth and yield [53]. Water organisms are also adversely affected by the Cu (II) ion in the water bodies including effects on an organism's survival, activity, growth, metabolism, and reproduction [54]. The characteristics of the treated wastewater fell short of the WHO standard [11]. The removal of Cu (II) ions in textile wastewater and the aqueous solution was the focus of this study. According to one study, the banana peel has a higher potential for removing Cu (II) ions from an aqueous solution [2].

#### 3.4.6. Adsorption Isotherms

**Figure 7:**
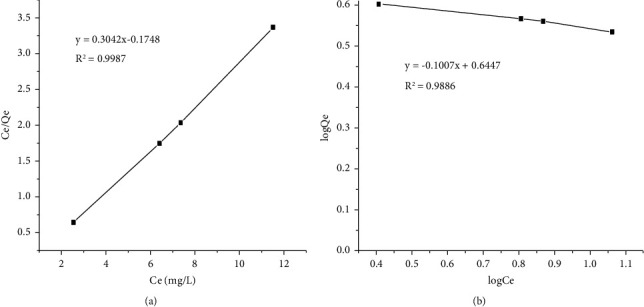
(a) Langmuir and (b) Freundlich adsorption isotherms for adsorption of Cu (II) on PBPA.

Adsorption isotherms describe the equilibrium relationships between the adsorbent and the adsorbate [2]. The equilibrium data obtained from the experiment for 1/qe vs. 1/Ce were shown in the Langmuir isotherm. The qm and *K*_ads_ constants were calculated, and the results are shown in Table 3 and Figure 7(a). The RL value for copper in this study was 1.74, which was larger than zero but less than unity, indicating that the equilibrium adsorption for both metal ions was favorable. The equilibrium data obtained for the Freundlich isotherm were plotted between log*q*_*e*_ and log*C*_*e*_. The constants *K*_*f*_ and 1/*n* were calculated, and the results are shown in Table 3 and Figure 7(b). The value of *n* for copper in this study was 2.7, which was larger than one but less than ten, indicating generally favorable chemical adsorption. The Langmuir model was shown to have greater correlation coefficient (*R*^2^) values, indicating that it was better suited to the experimental data. The Langmuir model was best for adsorption data though other models posed good association [2]. It was suggested that the adsorption of Cu (II) onto powdered banana peel follows Langmuir isotherm, which suggests that the adsorption takes place in the form of monolayer coverage on the surface of the powdered banana peel [55].

#### 3.4.7. Adsorption Kinetics

Kinetic studies are required for any type of biosorption process. Adsorption kinetics describes not only the mechanism of metal adsorption on adsorbents but also the rate of metal adsorption, which affects the metal contact time at the solid-liquid interface [2].

*Pseudo-First-Order*. The slopes of the plot of log (q_e_q_t_) vs. *t* as shown in equation (5) are obtained from the values of *k*_1_ shown in Table 4. The correlation coefficients (*R*^2^) for the pseudo-first-order kinetic model were low, indicating that the copper adsorption was significantly different from the pseudo-first-order kinetic model and could not explain the copper adsorption onto PBPWA.

*Pseudo-Second-Order*. The values of *k*_2_ were calculated using the intercepts of the plot *t*/qt vs. *t*. For all studied metal ion concentrations, the resulting correlation coefficients (*R*^2^) for the pseudo-second-order kinetic model were greater than 0.97, which is greater than that of the pseudo-first-order kinetic model. These findings revealed that the copper adsorption system investigated followed a pseudo-second-order kinetic model. These results indicated that the adsorption of Cu (II) onto powdered banana peel is more likely controlled by the chemisorption process that involved covalent forces through exchanging of electrons between adsorbent and adsorbate [46].

## 4. Conclusions

The physicochemical analysis of powdered banana peel showed that it has 3.8% moisture content, 3.5% ash content, 37.5% volatile matter, and 0.02 g/cm^3^ bulk density. The optimum condition of Cu (II) removal from synthetic wastewater on the powdered banana peel adsorbent was obtained at pH 5, adsorbent dose of 2 g/100 mL, initial concentration of 80 mg/L, and contact time of 90 min. Under the optimum conditions, the removal efficiency of copper from synthetic wastewater and real wastewater was 96.8% and 69.8%, respectively. The removal efficiency of Cu (II) ions in real textile wastewater was less than that of aqueous solution in the real wastewater, and other heavy metals and physicochemical parameters were also involved in the active sites of the adsorbent during the adsorption process. Based on the results obtained within the framework of this study, it appears that the powdered banana peel is a good adsorbent for removing copper (II) from textile industry wastewater. The results of the adsorption isotherm studies show that the Langmuir isotherm model provides a better fit for the adsorption process (*R*^2^ = 0.9987). From the adsorption kinetic study, the adsorption better follows the second-order kinetic model, indicating the chemical sorption to be the rate-limiting step. The results showed that banana peel waste could effectively absorb Cu (II) and the use of this low-cost agricultural waste in wastewater treatment applications is promising. Finally, banana peels, which are abandoned waste materials that may be found in abundance in various trash disposal sites, can be used to remove copper ions from textile wastewater.

## Figures and Tables

**Table 1:** Physicochemical characteristics of powdered banana peel.

Properties	Value
Ash content (%)	3.5
Fixed carbon (%)	55.23
Moisture content (%)	3.8
Bulk density (g/cc)	0.02
pH	6.55

**Table 2:** Textile industry wastewater (untreated and treated) characteristics.

Parameters	Unit	Untreated wastewater	Treated wastewater	Maximum limited value [11]
pH	pH unit	8.6	6.5	6.5–9.2
Temperature	^o^C	36.6	20.5	40
Turbidity	NUT	520	10	—
BOD_5_	mg/L	185.35	45	80
COD	mg/L	612.16	150	150
Cu (II)	mg/L	2.75	0.86	1.0
Cd (II)	mg/L	0.01	Null	0.1
Pb (II)	mg/L	2.02	0.268	0.1
Zn (II)	mg/L	1.7	0.55	5.0

**Table 3:** Langmuir and Freundlich isotherm model constants for copper onto PBPA.

Langmuir isotherm			Freundlich isotherm		
*Q*_*m*_ (mg/g)	*K*_*L*_ (L/mg)	*R* ^2^	*K*_*f*_	*N*	*R* ^2^
3.287	1.74	0.9987	0.6447	2.7	0.9889

**Table 4:** Pseudo-first-order and pseudo-second-order constants for Cu (II) adsorption on PBPWA.

Pseudo-first-order kinetics			Pseudo-second-order kinetics		
*q*_*e*_ (mg/g)	*K*_1_ (min^−1^)	*R* ^2^	*q*_*e*_ (mg/g)	*K*_2_ (min^−1^)	*R* ^2^
0.844	0.0243	0.98	5.741	0.0301	0.9913

## Data Availability

No data were used to support this study.

## Supplementary Materials

Supplementary Materials Figure 1: (a) banana peel waste and (b) banana peel powder.
