# Supplementary figures and images for: Isotherms and Kinetic Studies of Copper Removal from Textile Wastewater and Aqueous Solution Using Powdered Banana Peel Waste as an Adsorbent in Batch Adsorption Systems
Source: Int J Biomater. 2023 May 26;2023:2012069. doi: 10.1155/2023/2012069 (PMC10238140; doi:10.1155/2023/2012069)

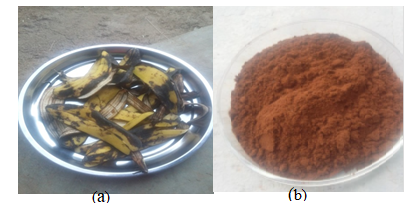


**Fig. 1. (a) Banana peel waste and (b) Banana peels powder.**

Supplement: Supplementary Materials — Figure 1: (a) banana peel waste and (b) banana peel powder. [file 2012069.f1.docx]
